# Silver Nanorings Fabricated by Glycerol-Based Cosolvent Polyol Method

**DOI:** 10.3390/mi11030236

**Published:** 2020-02-25

**Authors:** Zhihang Li, Dong Guo, Peng Xiao, Junlong Chen, Honglong Ning, Yiping Wang, Xu Zhang, Xiao Fu, Rihui Yao, Junbiao Peng

**Affiliations:** 1 Institute of Polymer Optoelectronic Materials and Devices, State Key Laboratory of Luminescent Materials and Devices, South China University of Technology, Guangzhou 510640, China; mslzhscut@mail.scut.edu.cn (Z.L.); msjlchen@gmail.com (J.C.); yyxlxz@126.com (X.Z.); 201630343721@mail.scut.edu.cn (X.F.); psjbpeng@scut.edu.cn (J.P.); 2 School of Medical Instrument & Food Engineering, University of Shanghai for Science and Technology, No.516 Jungong Road, Shanghai 200093, China; guodong99@tsinghua.org.cn; 3 School of Physics and Optoelectronic Engineering, Foshan University, Foshan 528000, China; xiaopeng@fosu.edu.cn; 4 State Key Laboratory of Mechanics and Control of Mechanical Structures, Nanjing University of Aeronautics and Astronautics, Nanjing 210016, China; yipingwang@nuaa.edu.cn; 5 Guangdong Province Key Lab of Display Material and Technology, Sun Yat-sen University, Guangzhou 510275, China

**Keywords:** wide bandgap semiconductors, flexible devices, silver nanoring, silver nanowire, polyol method, cosolvent

## Abstract

The urgent demand for transparent flexible electrodes applied in wide bandgap devices has promoted the development of new materials. Silver nanoring (AgNR), known as a special structure of silver nanowire (AgNW), exhibits attractive potential in the field of wearable electronics. In this work, an environmentally friendly glycerol-based cosolvent polyol method was investigated. The Taguchi design was utilized to ascertain the factors that affect the yield and ring diameter of AgNRs. Structural characterization showed that AgNR seeds grew at a certain angle during the early nucleation period. The results indicated that the yield and ring diameter of AgNRs were significantly affected by the ratio of cosolvent. Besides, the ring diameter of AgNRs was also tightly related to the concentration of polyvinylpyrrolidone (PVP). The difference of reducibility between glycerol, water, and ethylene glycol leads to the selective growth of (111) plane and is probably the main reason AgNRs are formed. As a result, AgNRs with a ring diameter range from 7.17 to 42.94 μm were synthesized, and the quantity was increased significantly under the optimal level of factors.

## 1. Introduction

With the development of flexible wide bandgap (WBG) semiconductor devices, such as flexible thin-film transistors (TFT) [1,2], wearable display devices [3,4], photovoltaic devices [5], and energy storage devices [6], the requirement for flexible electrodes, especially ones that are transparent and printable, has become urgent. As a traditional transparent conductive thin film (TCF), indium tin oxide (ITO) is brittle and requires high temperature preparation, which limits its application in flexible TCFs [7,8]. To meet such demand, several materials like graphene [9], carbon nanotubes [10], metal grid [11,12], and metal nanowires [13,14] have been studied over the years and achieved some substantial results.

Among these materials, silver nanowire (AgNW), known as the most suitable material to prepare TCFs, is considered to be a potential material because of its excellent electrical, optical, and mechanical properties as well as simple solution preparation [15,16,17,18,19]. Recently, a new form of silver nanostructure called silver nanoring (AgNR) was prepared as the by-product of AgNW. It showed high flexible performance with little effect on light transmission and also exhibited some attractive properties to be applied in optical nanoantennae, plasmonic devices, and optical manipulation [20,21]. Azani et al. simulated random networks deposited with conductive rings or wires and declared that AgNR can reach better optoelectrical property than AgNW at the same sheet resistance [22]. AgNR retains intrinsic chemical stability and low electrical resistivity like other silver materials and has strong localized surface plasmon resonance (LSPR) [23] to be used in energy conversion or signal enhancement. When AgNR and AgNW are deposited in flexible substrates, the two-dimensional structure of AgNR can increase the probability of overlapping to form a conductive network, and the maximum number of the contact point is twice as many as that of AgNW, which gives AgNR greater resistance against disconnect. At the same time, the ring shape gives the conductive network stronger tensile resistance [24]. Owing to these advantages, AgNR can be fabricated into printable flexible transparent electrodes to form WBG semiconductor devices with superior performance. However, like other nanorings composed of Cu [25], GaN [26], AlN [27], and ZnO [28], the preparation of AgNR still faces the difficulties of low yield and less purity, which seriously affect its application. Ethylene glycol (EG), the most commonly used solvent in the polyol method to prepare AgNWs, has subacute and chronic toxicity to humans. Glycerol, another polyol with higher hydroxyl content ratio, is far less harmful to humans and the environment and is commonly used in food and medicine. In this study, AgNRs were synthesized through an environmentally friendly glycerol-based cosolvent polyol method. The factors that influence the yield and average ring diameter were analyzed by the Taguchi design, and the growth mechanism was investigated by combining the results of structural characterization.

## 2. Materials and Methods 

The AgNRs were synthesized with a glycerol-based cosolvent polyol method. Polyvinylpyrrolidone (PVP, Shanghai Maclean Biochemical Co., Ltd., Shanghai, China) of different molecular weights (Mw) (K60, Mw ~360,000, K90 Mw ~1,300,000) was added in glycerol (Shanghai Richjoint Co., Ltd., Shanghai, China). The mixture was then heated to 160 °C with vigorous stirring. After 30 min, 26.88 mM NaCl (Shanghai Richjoint Co., Ltd.) or tetramethylammonium chloride (TMA-C, Shanghai Maclean Biochemical Co., Ltd., Shanghai, China) and 9 mM NaBr (Shanghai Richjoint Co., Ltd.) or tetrapropylammonium bromide (TPA-B, Shanghai Maclean Biochemical Co., Ltd.) dissolved in glycerol were added to the mixture and stirred for another 30 min. Next, 137.95 mM AgNO_3_ (Sinopharm Chemical Reagent Co., Ltd., Shanghai, China) dissolved in glycerol mixed with EG (Shanghai Richjoint Co., Ltd.) or deionized (DI) water was added dropwise to the mixture for 10 min. Then, the mixture was further kept at 160 °C for 1 h without stirring until the reaction finished. Under pressure conditions, the reaction was conducted in a hydrothermal kettle, and the reaction time was increased to 7 h. After cooling, the solution was purified with acetone and ethanol and suspended in ethanol. AgNR dispersion in ethanol was spin-coated on silicon wafers and then annealed at 130 °C for 15 min for characterization. SEM images were taken by Hitachi Regulus8100 field-emission SEM, and the quantity and ring diameter of silver nanorings were measured with Olympus 3D confocal laser scanning microscope (CLSM) OLS5000 (Olympus Corporation, Tokyo, Japan).

## 3. Results and Discussion

**Figure 1:**
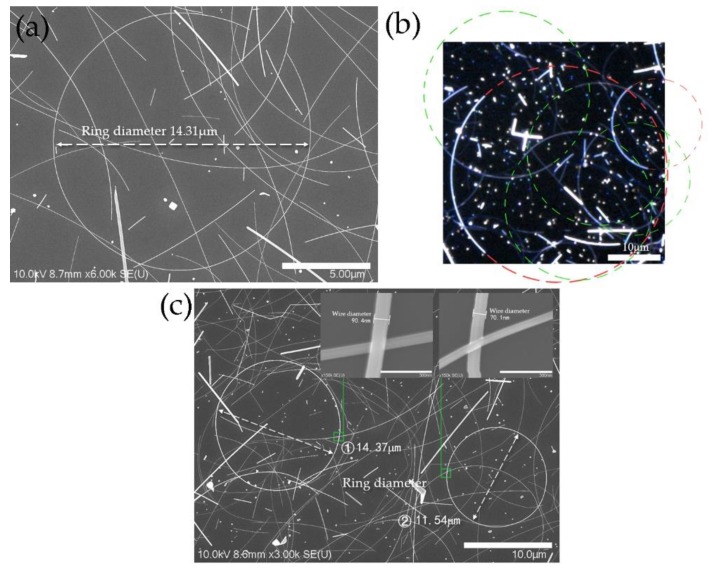
(**a**) SEM image of silver nanorings (AgNRs) before closure; (**b**) confocal laser scanning microscope (CLSM) image of silver nanoarcs; (**c**) SEM images of AgNRs after increasing reaction time to 1 h.

To fabricate AgNRs, a glycerol-based polyol method with a reaction time of 45 min was used. As shown in Figure 1a,b, AgNRs could hardly be observed except for several AgNRs before closure (Figure 1a) and some silver nanoarcs (Figure 1b). By completing the rest of the silver nanoarcs in Figure 1b, we could see that nearly all of them fitted well with the standard ring shape. This indicated that such arcs could grow into AgNRs if the reaction time was longer. The AgNRs seemingly grew along the track of the rings instead of becoming ring-shaped because of internal force after the two ends of AgNWs met. This suggests that AgNRs grow differently from AgNWs at the early stage, which implies that decahedral seeds [29] probably grow at a certain angle during the early nucleation period. To improve the yield of AgNRs, the reaction time was increased to 1 h. This resulted in AgNRs being synthesized with perfect geometry (Figure 1c), exhibiting the possibility of fabricating AgNRs with a higher yield.

To increase the number of decahedral seeds that grow along a certain angle and finally raise the yield of AgNRs, a cosolvent method was introduced to provide different nucleation effect and reduction potential [30]. EG, the most commonly used solvent in the polyol method [31], and water, the solvent for preparing AgNWs by the hydrothermal method [32], were chosen as other solvents with less unpredictable influence on the reaction. To further select the main factors that affect the quantity and average ring diameter of the prepared AgNRs, an L_8_(2^7^) orthogonal array was designed, and the corresponding results are presented in Table 1. The quantity of AgNRs was obtained by counting 50 images of 250 μm × 250 μm that were randomly selected by CLSM. The optimal level to be chosen and the rank of factors analyzed by the Taguchi design are listed in Table 2. The delta value in Table 2 represents the degree of influence on the result, and the rank values are the sequences of delta values. Both the concentration of PVP and the ratio of cosolvent strongly affected the quantity and average ring diameter, while the Mw of PVP and the type of chloride salt had less effect on the reaction. To increase the yield of AgNRs, the concentration of PVP, the ratio of cosolvent, and the type of cosolvent were chosen as the main factors for further experiment, while other factors were kept at the optimal level for greater quantity, as shown in Table 2. It is worth noting that the quantity listed in Table 1 is much less than that of our abovementioned work with a glycerol-based polyol method using NaCl, NaBr, and 82.8 mM PVP-K90 without any cosolvent and applying pressure. Among these factors, the addition of another solvent played an important role, as shown in Table 2, and a smaller ratio seemed to benefit the generation of AgNRs to some extent. Moreover, with excess addition of EG or DI water, the viscosity of the solution decreased sharply, which would suppress AgNWs from curving into the ring shape. Therefore, the ratio of cosolvent was reduced to ≈10% of the original.

**Figure 2:**
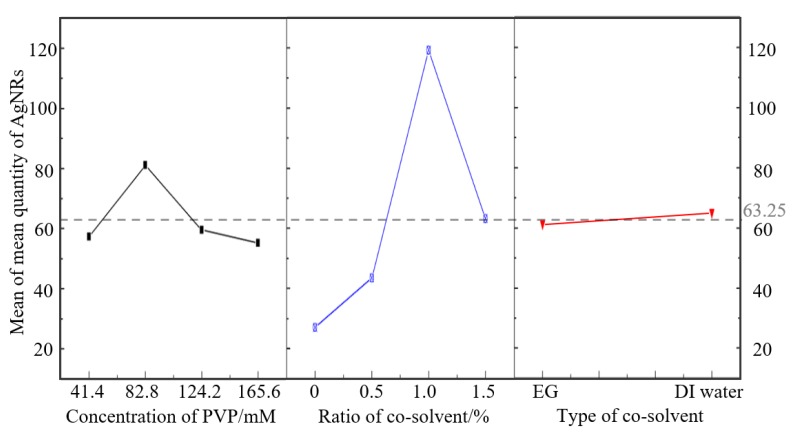
Plot of the main effects on the mean quantity of AgNRs.

Another L_16_(2^1^4^2^) orthogonal array was designed to study the optimal level of concentration of PVP, the ratio of cosolvent, and the type of cosolvent (Table 3). The analysis results for the quantity of AgNRs with the Taguchi design are shown in Figure 2 and Table 4. The mean quantity presented in Figure 2 was achieved by the following regression equation:

(1) $$\begin{array}{l} \mathrm{Quantity}\\ =\;63.25\;-\;6.0\;\mathrm{Concentration}\;\mathrm{of}\;\mathrm{PVP}\_1\;\\ +\;17.8\;\mathrm{Concentration}\;\mathrm{of}\;\mathrm{PVP}\_2\;\\ +\;3.8\;\mathrm{Concentration}\;\mathrm{of}\;\mathrm{PVP}\_3\;\\ -\;8.0\;\mathrm{Concentration}\;\mathrm{of}\;\mathrm{PVP}\_4\;\\ -\;36.3\;\mathrm{Ratio}\;\mathrm{of}\;\mathrm{cosolvent}/\%\_1\;\\ -\;19.7\;\mathrm{Ratio}\;\mathrm{of}\;\mathrm{cosolvent}/\%\_2\;\\ +\;56.0\;\mathrm{Ratio}\;\mathrm{of}\;\mathrm{cosolvent}/\%\_3\;\\ +\;0.0\;\mathrm{Ratio}\;\mathrm{of}\;\mathrm{cosolvent}/\%\_4\\ +\;1.88\;\mathrm{Type}\;\mathrm{of}\;\mathrm{cosolvent}\_1\\ +\;1.88\;\mathrm{Type}\;\mathrm{of}\;\mathrm{cosolvent}\_2 \end{array}$$

The number behind the factor refers to its level, and it was the value employed to substitute into the equation. According to Figure 2, the best level of above 3 factors was achieved by adding 1% DI water into glycerol and 82.8 mM PVP. Only the *p*-value of the ratio of cosolvent was less than 0.05, which meant the ratio of cosolvent had a significant effect on the quantity. The mean quantity rose slightly from 27.00 to 43.50 as 0.5% cosolvent was added and then rose significantly to 119.25 when the ratio was 1%; it finally rapidly declined to 63.25. Despite the significant effect of ratio, the mean quantity hardly changed with a delta value of merely 3.75 when EG was replaced with DI water. Similarly, with the increase in concentration of PVP, the mean quantity rose slightly and reached a peak of 81.00 at 82.8 mM, then eventually decreased to 55.25. 

Correspondingly, the mean ring diameter was analyzed by the following regression equation:

(2) $$\begin{array}{l} \mathrm{Ring}\;\mathrm{diameter}\;\\ =\;15.441\;\\ -\;1.546\;\mathrm{Concentration}\;\mathrm{of}\;\mathrm{PVP}\_1\;\\ +\;0.389\;\mathrm{Concentration}\;\mathrm{of}\;\mathrm{PVP}\_2\;\\ -\;0.499\;\mathrm{Concentration}\;\mathrm{of}\;\mathrm{PVP}\_3\;\\ -\;0.657\;\mathrm{Concentration}\;\mathrm{of}\;\mathrm{PVP}\_4\;\\ -\;0.671\;\mathrm{Ratio}\;\mathrm{of}\;\mathrm{cosolvent}/\%\_1\;\\ -\;0.662\;\mathrm{Ratio}\;\mathrm{of}\;\mathrm{cosolvent}/\%\_2\;\\ +\;0.937\;\mathrm{Ratio}\;\mathrm{of}\;\mathrm{cosolvent}/\%\_3\;\\ +\;0.928\;\mathrm{Ratio}\;\mathrm{of}\;\mathrm{cosolvent}/\%\_4\;\\ -\;0.208\;\mathrm{Type}\;\mathrm{of}\;\mathrm{cosolvent}\_1\;\\ +\;0.208\;\mathrm{Type}\;\mathrm{of}\;\mathrm{cosolvent}\_2 \end{array}$$

**Figure 3:**
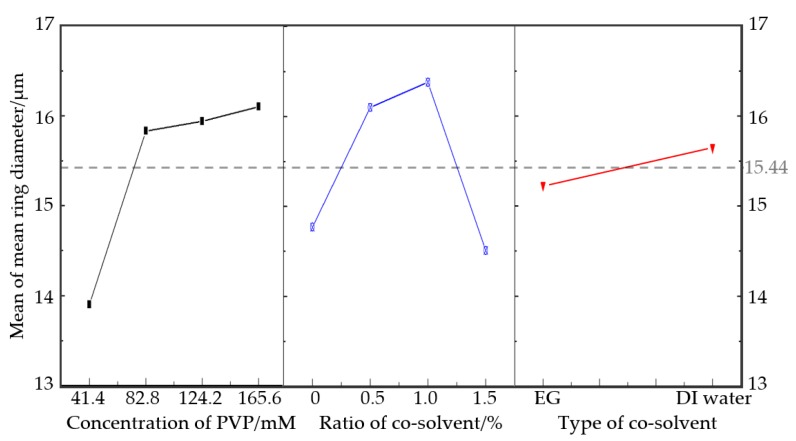
Plot of the main effects for mean ring diameter.

As can be seen from the results in Figure 3 and Table 5, the optimal level was 165.6 mM PVP and the addition of 1% DI water into glycerol. Like the quantity, the type of cosolvent had little to do with the mean ring diameter. However, the *p*-value of both the ratio of cosolvent and concentration of PVP was less than 0.05, showing a significant effect. However, the mean ring diameter rose more gently as the ratio of cosolvent increased. It rose greatly when the concentration of PVP increased to 82.8 mM, then rose mildly as more PVP was introduced. Putting the two figures together, the peak of the ratio of cosolvent appeared at 1%, and no matter how much EG or DI water was added, both of them produced a noticeable effect without much difference.

**Figure 4:**
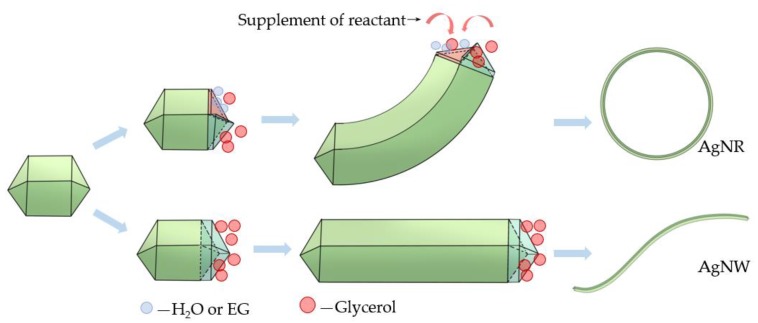
Schematic of the growth of AgNR with glycerol-based cosolvent polyol method.

The EG or DI water added to glycerol to form the cosolvent had some features distinct from glycerol. To explain the mechanism of the effect of the cosolvent on the synthesis of AgNRs, a schematic is illustrated in Figure 4. On the one hand, EG and water were less reductive than glycerol. At the early nucleation period of AgNRs, due to the concentration fluctuation, a small amount of EG or water adsorbed on several (111) planes of decahedral seeds diluted the concentration of glycerol in the micro area, which provided slower growth rate than those reduced by glycerol on these planes. As a result, these seeds grew with a certain angle. Meanwhile, EG or water on these planes was localized on account of the supplement of the reactant. Therefore, the growth direction of AgNRs was maintained, and the two ends finally met and combined to become rings. When excess EG or water was added, the difference between the planes became smaller because the concentration of each area was similar, resulting in the growth of AgNWs instead of AgNRs [33]. On the other hand, the viscosity of EG and water is far less than that of glycerol. Although glycerol has stronger reducibility, its high viscosity slowed down the migration velocity, resulting in an incomplete reaction. By adding an appropriate ratio of EG or water, the viscosity could fall to a more suitable level [34]. What is more, they could serve as the medium to promote the proton hopping on the surface of the reactant, facilitating the charge transfer process [35]. The cosolvent system also provided better solubility for AgNO_3_ than glycerol. All these features increased the ion mobility in the solution and then facilitated the process of reaction. However, when the ratio was too high, the condition beneficial for the growth of AgNRs provided by glycerol was disturbed, causing a reduction of AgNRs. 

**Figure 5:**
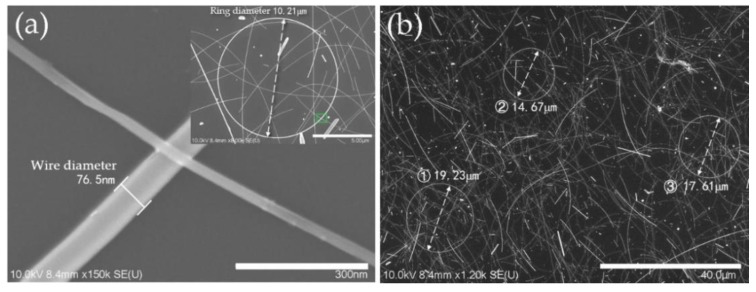
SEM images of AgNRs synthesized with the optimal level for greater quantity of AgNRs at high (**a**) and low (**b**) magnification.

**Figure 6:**
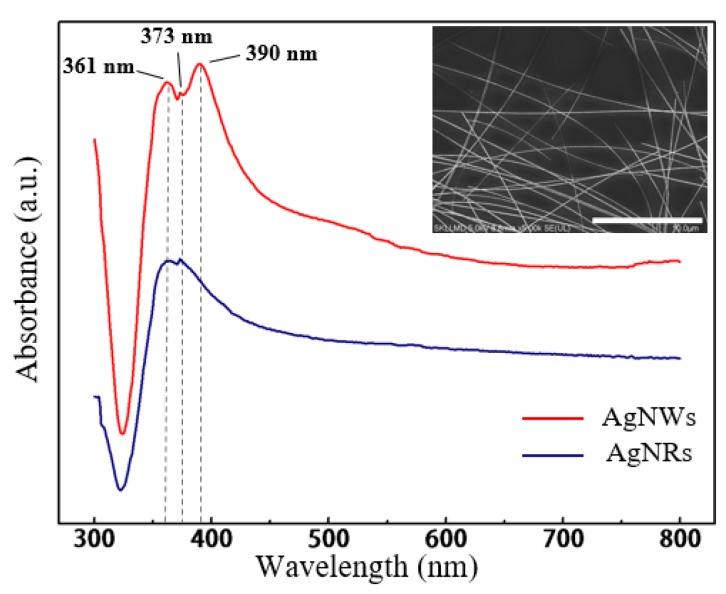
UV−vis adsorption spectra of AgNRs and silver nanowires (AgNWs). The inset is the SEM image of AgNWs.

In the case of added PVP, which acts as the capping agent [36], when the concentration increased to 82.8 mM, the effect of restricting the wire diameter by covering the surface of AgNRs became significant. More early seeds were controlled to grow into AgNRs or AgNWs, resulting in higher precursor utilization. In other words, AgNRs with larger ring diameter and higher yield tended to be generated. With excess PVP, such capping effect was almost saturated [37], showing that the ring diameter only slightly increased. Besides, as a material that easily absorbs moisture, excess PVP would absorb the water or EG molecule and restrict it from diffusion, then nullify the cosolvent effect mentioned above, leading to a low yield of AgNRs. With the optimal level of factors for a greater quantity of AgNRs (same as Sample 15), AgNRs were synthesized with ring diameter ranging from 7.17 to 42.94 μm and wire diameter of about 76 nm, as shown in Figure 5, and the quantity increased significantly under such conditions. Figure 6 compares the UV−vis adsorption spectra of AgNRs and AgNWs; the AgNWs here were synthesized using EG as solvent. As can be seen, the adsorption spectra of both materials had two surface plasmon resonance (SPR) peaks at ~361 nm and ~373 nm, but the spectra of AgNWs had another peak around ~390 nm with high intensity, which corresponded to transverse plasmon resonance [38,39]. Such a strong absorption peak at ~390 nm could influence the optical properties of the film; hence, AgNRs thin films have the potential to substitute AgNWs as TCF materials. This work provides a new, environmentally friendly method to fabricate AgNRs with a solvent consisting of glycerol and water instead of EG [40]. This makes the synthesis of AgNRs a different process from the former method, which is a by-product of AgNWs [20,21].

## 4. Conclusions 

In conclusion, we have reported a modified, environmentally friendly polyol method to synthesize AgNRs with higher yield using a cosolvent consisting of glycerol and DI water, with other factors determined by employing the Taguchi design. Structural characterization revealed that AgNRs grew along the track of rings from the early stage of the reaction. To achieve high yield of AgNRs, the addition of DI water or EG was significant, which acted as the medium to increase ion mobility and another reductant that is less reductive to control the growth direction. Otherwise, the ratio of it should be precisely controlled. As a result, AgNRs with ring diameter ranging from 7.17 to 42.94 μm and wire diameter of about 76 nm were synthesized with higher yield. The research indicates the potential to synthesize pure AgNRs and to further apply them in flexible WBG semiconductor devices.

## Figures and Tables

**Table 1:** L_8_(2^7^) orthogonal array and the corresponding result.

Sample	Mw of PVP	Concentration of PVP /mM	Chloride Salt	Bromide Salt	Type of Cosolvent	Ratio of Cosolvent/%	Pressure	Quantity of AgNRs	Average Ring Diameter/μm
1	K90	41.4	Na^+^	Na^+^	EG	5	with	28	14.77
2	K90	41.4	Na^+^	TPA-	DI	10	without	5	10.38
3	K90	82.8	TMA-	Na^+^	EG	10	without	76	14.65
4	K90	82.8	TMA-	TPA-	DI	5	with	75	14.80
5	K60	41.4	TMA-	Na^+^	DI	5	without	24	13.25
6	K60	41.4	TMA-	TPA-	EG	10	with	10	12.40
7	K60	82.8	Na^+^	Na^+^	DI	10	with	14	15.00
8	K60	82.8	Na^+^	TPA-	EG	5	without	108	14.90

**Table 2:** The optimal level and the rank of factors (larger the better).

Results	Factors	Mw of PVP	Concentration of PVP/mM	Chloride Salt	Bromide Salt	Type of Cosolvent	Ratio of Cosolvent/%	Pressure
Quantity of AgNRs	Level	K90	82.8	TMA-	TPA-	EG	5	without
	Delta	7.00	51.50	7.50	14.00	26.00	32.50	21.50
	Rank	7	1	6	5	3	2	4
Average ring diameter	Level	K60	82.8	TMA-	TPA-	DI	5	with
	Delta	0.24	2.14	0.01	1.30	0.82	1.32	0.95
	Rank	6	1	7	3	5	2	4

**Table 3:** L_16_(2^1^4^2^) orthogonal array and the corresponding result.

Sample	Concentration of PVP/mM	Ratio of Cosolvent/%	Type of Cosolvent	Quantity	Average Ring Diameter/μm
9	41.4	0	EG	32	12.80
10	41.4	0.5	EG	18	13.47
11	41.4	1	DI	140	15.74
12	41.4	1.5	DI	39	13.57
13	82.8	0	EG	42	15.18
14	82.8	0.5	EG	65	17.15
15	82.8	1	DI	162	16.56
16	82.8	1.5	DI	55	14.43
17	124.2	0	DI	27	15.45
18	124.2	0.5	DI	41	16.31
19	124.2	1	EG	97	16.72
20	124.2	1.5	EG	73	15.28
21	165.6	0	DI	7	15.65
22	165.6	0.5	DI	50	17.48
23	165.6	1	EG	78	16.49
24	165.6	1.5	EG	86	14.77

**Table 4:** Analysis of variance with the general linear model for the quantity of AgNRs.

Source	DF	Adj SS	Adj MS	Delta	*F*-Value	*p*-Value
Concentration of PVP	3	1716.5	572.17	25.75	0.79	0.531
Ratio of cosolvent	3	19360.5	6453.5	92.25	8.96	0.006
Type of cosolvent	1	56.3	56.25	3.75	0.08	0.787
Error	8	5761.7	720.22	−	−	−
Total	15	26895.0	−	−	−	−

**Table 5:** Analysis of variance with the general linear model for ring diameter.

Source	DF	Adj SS	Adj MS	Delta	*F*-Value	*p*-Value
Concentration of PVP	3	12.8857	4.2952	2.20	9.05	0.006
Ratio of cosolvent	3	10.5079	3.5026	1.86	7.38	0.011
Type of cosolvent	1	0.6931	0.6931	0.42	1.46	0.261
Error	8	3.7951	0.4744	−	−	−
Total	15	27.8817	−	−	−	−
